# The hospital Israelita Albert Einstein standards for constitutional sequence variants classification: version 2023

**DOI:** 10.1186/s40246-023-00549-6

**Published:** 2023-11-16

**Authors:** Caio Robledo D’Angioli Costa Quaio, José Ricardo Magliocco Ceroni, Michele Araújo Pereira, Anne Caroline Barbosa Teixeira, Renata Yoshiko Yamada, Vivian Pedigone Cintra, Eduardo Perrone, Marina De França, Kelin Chen, Renata Moldenhauer Minillo, Cheysa Arielly Biondo, Mariana Rezende Bandeira de Mello, Lais Rodrigues Moura, Amanda Thamires Batista do Nascimento, Karla de Oliveira Pelegrino, Larissa Barbosa de Lima, Luiza do Amaral Virmond, Carolina Araujo Moreno, Joana Rosa Marques Prota, Jessica Grasiela de Araujo Espolaor, Thiago Yoshinaga Tonholo Silva, Gabriel Hideki Izuka Moraes, Gustavo Santos de Oliveira, Livia Maria Silva Moura, Marcel Pinheiro Caraciolo, Rafael Lucas Muniz Guedes, Michel Chieregato Gretschischkin, Pedro Lui Nigro Chazanas, Carolina Naomi Izo Nakamura, Rodrigo de Souza Reis, Carmen Melo Toledo, Fernanda Stussi Duarte Lage, Giovanna Bloise de Almeida, José Bandeira do Nascimento Júnior, Milena Andreuzo Cardoso, Victor de Paula Azevedo, Tatiana Ferreira de Almeida, Murilo Castro Cervato, Joao Bosco de Oliveira Filho

**Affiliations:** 1 Laboratório Clínico, Hospital Israelita Albert Einstein, Av. Albert Einstein 627, São Paulo, SP CEP 05652-000 Brazil; 2 VarsOmics, Hospital Israelita Albert Einstein, São Paulo, SP Brazil

**Keywords:** Variant classification, Sequence variant, Genetic testing, Genomics, Guideline, Brazil

## Abstract

**Background:**

Next-generation sequencing has had a significant impact on genetic disease diagnosis, but the interpretation of the vast amount of genomic data it generates can be challenging. To address this, the American College of Medical Genetics and Genomics and the Association for Molecular Pathology have established guidelines for standardized variant interpretation. In this manuscript, we present the updated Hospital Israelita Albert Einstein Standards for Constitutional Sequence Variants Classification, incorporating modifications from leading genetics societies and the ClinGen initiative.

**Results:**

First, we standardized the scientific publications, documents, and other reliable sources for this document to ensure an evidence-based approach. Next, we defined the databases that would provide variant information for the classification process, established the terminology for molecular findings, set standards for disease-gene associations, and determined the nomenclature for classification criteria. Subsequently, we defined the general rules for variant classification and the Bayesian statistical reasoning principles to enhance this process. We also defined bioinformatics standards for automated classification. Our workgroup adhered to gene-specific rules and workflows curated by the ClinGen Variant Curation Expert Panels whenever available. Additionally, a distinct set of specifications for criteria modulation was created for cancer genes, recognizing their unique characteristics.

**Conclusions:**

The development of an internal consensus and standards for constitutional sequence variant classification, specifically adapted to the Brazilian population, further contributes to the continuous refinement of variant classification practices. The aim of these efforts from the workgroup is to enhance the reliability and uniformity of variant classification.

**Supplementary Information:**

The online version contains supplementary material available at 10.1186/s40246-023-00549-6.

## Background

Next-generation sequencing (NGS) has revolutionized the molecular diagnosis of patients with genetic diseases. However, the enormous amounts of genomic data generated by NGS present significant challenges in data interpretation. As a result, sophisticated analysis and interpretation methods are essential for achieving accurate and reliable molecular diagnosis.

Variant classification is a critical, dynamic, and systematic process that involves collecting evidence from multiple sources, such as scientific literature, control databases, and in silico predictors [1]. The primary goal of this process is to interpret genetic findings accurately. Aiming to standardize the process for variant classification, the American College of Medical Genetics and Genomics (ACMG), in conjunction with the Association for Molecular Pathology (AMP), created guidelines for variant interpretation based on specific criteria and evidence types [2]. The majority of laboratories worldwide have adopted the ACMG/AMP guidelines for 1) criteria direction (benign [B] or pathogenic [Pt]) and 2) the levels of strength for evidence (stand-alone [A], very strong [VS], strong [S], moderate [M], or supporting [P]) to classify variants [3]. This approach has been demonstrated to be a key step toward achieving a more uniform classification process in variant interpretation.

The ACMG/AMP guidelines have undergone a series of modifications and updates since their release in 2015, which have been proposed by various genetics societies and initiatives. While the five categories for variant classification have remained unchanged (pathogenic, likely pathogenic, uncertain significance, likely benign, and benign), the guidelines have been through several disease-focused specifications and refinements in some criteria strengths [3, 4]. These modifications have been introduced to keep pace with the rapidly evolving field of genetics and to improve the consistency of variant interpretation, although they have also increased the complexity of the process.

Recently, a Bayesian statistical reasoning approach has been proposed to translate the ACMG/AMP guidelines into a Bayesian framework for variant interpretation [5, 6]. This model enables inferences about the molecular impact of variants according to current data using probability theory. The model maps the four levels of evidence (P, M, S, and VS) to exponentially scaled odds of pathogenicity, which are 2.08:1, 4.33:1, 18.7:1, and 350:1, respectively. This approach provides a more quantitative and probabilistic interpretation of variant evidence, which can improve the precision of variant classification.

This manuscript presents the most recent version of the Standards for Constitutional Sequence Variants Classification of The Hospital Israelita Albert Einstein (HIAE), located in São Paulo, Brazil. These standards have been updated to incorporate the modifications proposed by leading genetics societies and the ClinGen initiative.

## Results

### Sources

Table 1 presents a comprehensive summary of the scientific publications, documents, and other sources that were gathered by our team to develop this standardized evidence-based framework for classification of sequencing variants.

**Table 1:** Summary of sources used to develop the Hospital Israelita Albert Einstein Standards for Constitutional Sequence Variants Classification

Criteria	Document	Source	Year
All	Richards S, et al. *Standards and guidelines for the interpretation of sequence variants: a joint consensus recommendation of the American College of Medical Genetics and Genomics and the Association for Molecular Pathology*. Genet Med. 2015;17:405–424	PMID: 25741868 [2]	2015
	Harrison SM, et al. *Overview of Specifications to the ACMG/AMP Variant Interpretation Guidelines*. Curr Protoc Hum Genet. 2019;103:e93	PMID: 31479589 [3]	2019
	Biesecker LG, Harrison SM. *ClinGen Sequence Variant Interpretation Working Group. The ACMG/AMP reputable source criteria for the interpretation of sequence variants*. Genet Med. 2018 Dec;20(12):1687–1688	PMID: 29543229 [7]	2018
	Tavtigian SV, et al. *Modeling the ACMG/AMP variant classification guidelines as a Bayesian classification framework*. Genet Med. 2018;20:1054–1060	PMID: 29300386 [5]	2018
	Tavtigian SV, et al. *Fitting a naturally scaled point system to the ACMG/AMP variant classification guidelines*. Hum Mutat. 2020;41:1734–1737	PMID: 32720330 [6]	2020
	ClinGen General Sequence Variant Curation Process	clinicalgenome.org/site/assets/files/3677/clingen_variant-curation_sopv1.pdf	2019
	ACGS Best Practice Guidelines for Variant Classification in Rare Disease 2020	www.acgs.uk.com/media/11631/uk-practice-guidelines-for-variant-classification-v4-01-2020.pdf	2020
	Tavtigian SV, et al. *Fitting a naturally scaled point system to the ACMG/AMP variant classification guidelines*. Hum Mutat. 2020;41:1734–1737	PMID: 32720330 [6]	2020
	ClinGen Variant Curation Standard Operating Procedure, Version 2	clinicalgenome.org/docs/variant-curation-standard-operating-procedure-version-2/	2021
	ClinGen Variant Curation Standard Operating Procedure, Version 3	clinicalgenome.org/docs/variant-curation-standard-operating-procedure-version-3/	2022
	ClinGen Sequence Variant Interpretation Working Group	clinicalgenome.org/working-groups/sequence-variant-interpretation/	Dynamic
PVS1	Abou Tayoun AN, et al. *Recommendations for interpreting the loss of function PVS1 ACMG/AMP variant criterion*. Hum Mutat. 2018;39(11):1517–1524	PMID: 30192042 [8]	2018
PS3/BS3	Brnich SE, et al. *Recommendations for application of the functional evidence PS3/BS3 criterion using the ACMG/AMP sequence variant interpretation framework*. Genome Med. 2019;12(1):3	PMID: 31892348 [9]	2019
PP3/BP4/BP7	Cooper GM, et al. *Distribution and intensity of constraint in mammalian genomic sequence*. Genome Res. 2005;15(7):901–913	PMID: 15965027 [10]	2005
	Jian X, et al. In silico* prediction of splice-altering single nucleotide variants in the human genome*. Nucleic Acids Res. 2014;42:13,534–13,544	PMID: 25416802 [11]	2014
	Ghosh R, et al. *Evaluation of *in silico* algorithms for use with ACMG/AMP clinical variant interpretation guidelines*. Genome Biol. 2017;18(1):225	PMID: 29179779 [12]	2017
	Tian Y, et al. *REVEL and BayesDel outperform other *in silico* meta-predictors for clinical variant classification*. Sci Rep. 2019;9:12,752	PMID: 31484976 [13]	2019
	Jaganathan K, et al. *Predicting Splicing from Primary Sequence with Deep Learning*. Cell. 2019;176(3):535–548.e24	PMID: 30661751 [14]	2019
	Pejaver V, et al. *Calibration of computational tools for missense variant pathogenicity classification and ClinGen recommendations for PP3/BP4 criteria*. Am J Hum Genet. 2022;109:2163–2177	PMID: 36413997 [15]	2022
BA1	Ghosh R, et al. *Updated recommendation for the benign stand-alone ACMG/AMP criterion*.Hum Mutat. 2018;39:1525–1530	PMID: 30311383 [16]	2018
PP2/PM1	Walsh R, et al. *Quantitative approaches to variant classification increase the yield and precision of genetic testing in Mendelian diseases: the case of hypertrophic cardiomyopathy*. Genome Med. 2019;11:5	PMID: 30696458 [17]	2019

The first column outlines the criteria evaluated by each document, while the second and third columns provide information about each source. The final column indicates the year of publication for each document

### Variant databases

Our service utilizes several variant databases as a source of molecular, epidemiologic, and clinical information. These databases are widely recognized as valuable resources for the classification and interpretation of sequence variants and contain extensive information on the clinical significance, frequencies in different populations, and functional impact of known variants.

Databases for population frequencies include the Genome Aggregation Database (gnomAD) [18] and the Online Archive of Brazilian Mutations (ABraOM) [19]. Population frequency criteria are applied for allele frequencies in overall populations and subpopulations of gnomAD (African/African American (AFR); American Admixed/Latino (AMR); East Asian (EAS); Non-Finnish European (NFE); South Asian (SAS)) with no founder effect, more than 2,000 alleles tested and variants present in 5 alleles in the databases.

Databases for pathogenic variants and phenotypic and genotypic data include ClinVar [20]; Single-Nucleotide Polymorphism Database (dbSNP) [21]; Database of Chromosomal Imbalance and Phenotype in Humans using Ensembl Resources (DECIPHER); Online Mendelian Inheritance in Man (OMIM); Clinical Genome Resource (ClinGen) [4, 22, 23]; published articles; and our internal database, which currently includes genomic information from 13,609 exomes, 3,706 genomes and 2,737 targeted-gene panels.

### Terminology of molecular findings

The workgroup adopted the specific standard terminology proposed by ACMG/AMP for variant classification, including the terms 'pathogenic', 'likely pathogenic', 'variant of uncertain significance' (VUS), 'likely benign', and 'benign', to describe variants identified in Mendelian disorders.

### Disease-Gene association and clinical impact of genes

Our service uses a systematic approach to collect and evaluate available scientific evidence to establish the clinical validity of gene-disease associations for all genes. To achieve this, we followed the gene-disease classification framework proposed by ClinGen, including “limited”, “moderate”, “strong”, and “definite” evidence, and classified genes with contradictory evidence as “disputed” or “refuted” [24]. In cases where ClinGen had already curated a gene, we utilized their classification as a standard. For genes not previously classified by ClinGen, our team followed the same approach to curate the gene-disease association validity.

Overall, variant classification is applied only for those genes whose clinical validity is defined as at least limited by the ClinGen group or our internal assessment. Additionally, the molecular impact of a variant and its consequent classification are assessed according to its effect on the primary transcript, which is prioritized in our service in the following order of transcript references: MANE Select, RefSeq Select, MANE Clinical Plus, and RefSeq. If only RefSeq transcripts are described, the largest transcript is chosen as the primary transcript.

### Criteria nomenclature

**Fig. 1:**
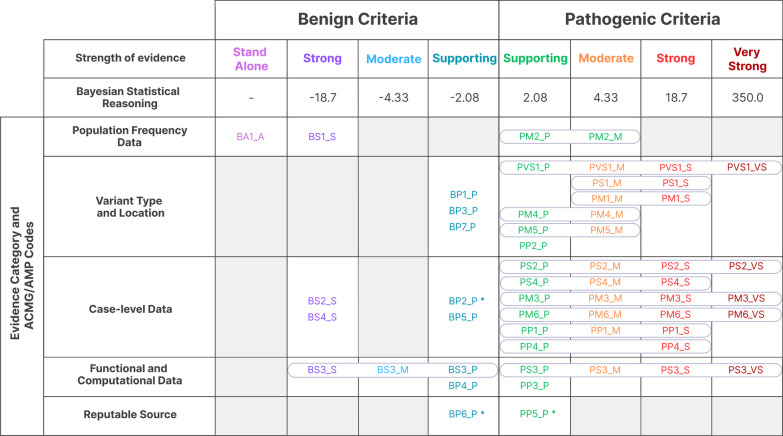
Criteria categories and their strength levels for the classification and interpretation of sequence variants. This scheme shows the five categories of all criteria, their direction (benign or pathogenic), and the corresponding strength. The third line shows the scaled odds of pathogenicity using the Bayesian statistical reasoning approach [5]. Pathogenic criteria include PVS1, PS1–4, PM1–6, and PP1–5, while benign criteria include BA1, BS1–4, and BP1–6. Each criterion is followed by possible weight modifications (stand-alone [A], very strong [VS], strong [S], moderate [M], or supporting [P]). Criteria marked with “*” are not used by our team. Adapted from Harrison et al. [3]

These standards have adopted the use of two sets of criteria originally proposed by the ACMG/AMP: (1) pathogenic criteria include PVS1, PS1–4, PM1–6 and PP1–5; (2) benign criteria include BA1, BS1–4 and BP1–6. These criteria are divided into five categories, namely population frequency data, variant type and location, case-level data, functional and computational data, and renewable source [3]. The weight for each criterion was modified based on the latest evidence from the literature and professional judgment. A comprehensive summary of all criteria and their categories and weights is presented in Fig. 1.

### Genes with specific criteria modulation and classification defined by variant curation expert panels (VCEPs)

The classification process for numerous genes involves specific rules curated by VCEPs. Our service strictly adheres to the criteria modulation and variant classification workflow for all genes curated by their respective VCEP. The lists of all genes curated by a VCEP and their gene-specific rules are compiled in the ClinGen CSpec Registry UI, a centralized database storing approved Criteria Specifications from VCEPs in a structured, machine-readable format. The latest version of the CSpec Registry can be accessed online at https://cspec.genome.network/cspec/ui/svi.

### Cancer genes

The process of criteria modulation and variant classification for cancer genes has particular specifications that differ from our general rules. These differences are described below. The list of cancer genes to which these specifications apply is compiled in Additional file 1: Table S1. It is important to note that our internal specifications for cancer genes do not apply to genes curated by VCEPs, including the *APC*, *ATM*, *CDH1*, *DICER1*, *PALB2*, *PIK3CA*, *PTEN*, *RUNX1*, and *TP53* genes. For these genes, our service strictly follows the criteria modulation and variant classification workflow curated by their respective VCEP, as stated above.

### The hospital Israelita Albert Einstein standards for Constitutional sequence variants classification: version 2023

Below, we outline our general rules for classifying sequence variants. It is worth mentioning again that these general rules do not apply to genes curated by VCEPs, as they follow their own specific workflow, which is not entirely covered in this article. Additionally, we provide specific modulations for cancer genes.

Our standards are based on the ACMG/AMP guidelines, and we have retained the original nomenclature for each criterion followed by its corresponding weight modulation. For clarity, we first provide the original nomenclature of each criterion, its definition (D), any possible weight modulations (W), conflicts with other criteria (C) and internal modifications and adaptations (MA) that have been incorporated into our standards.

#### Population frequency data

- **1A) BA1**
  - D: Allele frequency is above 5%.
  - W: BA1_A
  - C: BA1, BS1 and PM2 are mutually exclusive.
  - MA: This represents the only criterion that can be used as a single piece of evidence to classify a variant as benign according to allele frequencies from the databases previously listed. The BA1_A criterion is employed if the variant is not included in the ClinGen exception list for BA1 (clinicalgenome.org/site/assets/files/3460/ba1_exception_list_07_30_2018.pdf) according to the following requirements:
    1. For a subset of 61 genes with specific recommendations from ClinGen, the frequency thresholds are specified in Additional file 1: Table S2 [16].
    2. For the remaining genes, BA1_A is applied for an allele frequency ≥ 0.05.

- **1B) BS1**
  - D: Allele frequency is greater than expected for the disorder.
  - W: BS1_S
  - C: BS1, BA1 and PM2 are mutually exclusive.
  - MA: We have used a conservative approach for the general use of this criterion. The requirements for the use of BS1 and its corresponding weight are as follows:
    1. For a subset of 61 genes with specific recommendations from ClinGen, the frequency thresholds are specified in Additional file 1: Table S2.
    2. For the remaining genes, BS1_S is used if the allele frequency is ≥ 0.1% for AD or ≥ 1% for AR, AD/AR or XL (Table 2).

**Table 2:** Frequency thresholds for the BS1_S, PM2_P and PM2_M criteria

Gene category	Inheritance	Criteria		
		BS1_S	PM2_P	PM2_M
Cancer genes	AD	> 0.1%	< 0.004%	absent
	AR, AD/AR or XL	> 1%	< 0.04%	< 0.004%
Remaining genes	AD	> 0.1%	< 0.001%	absent
	AR, AD/AR or XL	> 1%	< 0.01%	< 0.001%

- **1C) PM2**
  - D: Absent from controls or at an extremely low frequency if recessive
  - W: PM2_P; PM2_M
  - C: PM2, BA1 and BS1 are mutually exclusive
  - MA:﻿ The PM2 criterion is used according to the following requirements (also shown in Table 2):
    1. For a subset of 61 genes with specific recommendations from ClinGen, the thresholds of PM2 and its corresponding weight are specified in Additional file 1: Table S2.
    2. For cancer genes (Additional file 1: Table S1), PM2_M is used when the variant is absent in AD conditions or with a frequency < 0.004% in AR, AD/AR or XL conditions; PM2_P is used when PM2_M conditions are not met and the allele frequency is < 0.004% for AD or < 0.04% for AR, AD/AR or XL.
    3. For the remaining genes, PM2_M is applied when the variant is absent from controls for AD conditions or at a frequency < 0.001% for AR, AD/AR or XL conditions; PM2_P is used when PM2_M conditions are not met and the allele frequency is < 0.001% for AD or < 0.01% for AR, AD/AR or XL (Table 2).

#### Variant type and location

- **2A) BP1**
  - D: Missense variant in a gene for which primarily truncating variants are known to cause disease
  - W: Not applied, except for curated variants from a recognized VCEP
  - MA:﻿ Due to the challenges associated with determining the deleterious effects of rare missense variants that have not been subjected to functional studies or are not located within critical domains, our group has decided not to apply this criterion at present.

- **2B) BP3**
  - D: In-frame deletions/insertions in a repetitive region without a known function.
  - W: BP3_P
  - C: BP3, PM4 and PVS1 are mutually exclusive.
  - MA: Due to the inherent difficulties in accurately defining repetitive regions and critical domains, particularly in genes with limited evaluation, our group has currently chosen not to routinely employ this criterion. However, in exceptional cases where compelling evidence is available, the use of this criterion may be considered.

- **2C) BP7**
  - D: A synonymous (silent) variant for which splicing prediction algorithms predict no impact on the splice consensus sequence or the creation of a new splice site, and the nucleotide is not highly conserved
  - W: BP7_P
  - C: PP3, BP4 and BP7 are mutually exclusive
  - MA:﻿ Our current approach for applying the BP7 criterion involves utilizing a combination of a SpliceAI score ≤ 0.2 and a GERP score < 0.

- **2D) PP2**
  - D: Missense variant in a gene that has a low rate of benign missense variation and in which missense variants are a common mechanism of disease
  - W: PP2_P
  - C: PM1 and PP2 may overlap, and their simultaneous use requires caution
  - MA:﻿ The PP2_P criterion is used when both of the following requirements are met, based on the gnomAD missense constraint Z score:
    1. The gene has at least three previously reported pathogenic missense variants.
    2. The gnomAD missense constraint Z score for the region harboring the variant is > 3.09 [25].

- **2E) PM1**
  - D: Located in a mutational hot spot and/or critical and well-established functional domain (e.g., active site of an enzyme) without benign variation
  - W: PM1_M; PM1_S
  - C: PM1 and PP2 may overlap, and their simultaneous use requires caution; PM1 and PM5 may overlap, and their simultaneous use requires caution
  - MA: The PM1 criterion is used according to the following requirements, based on the DECIPHER database:
    1. PM1_S for cysteine substitutions that result in an uneven number of cysteine residues within an EGF-like repeat in *NOTCH3*.
    2. PM1_S for glycine substitutions in *COL1A1* or other collagen genes.
    3. PM1_S for cysteine or histidine substitutions in C2H4 zinc fingers (such as *GLI3*).
    4. PM1_M for substitutions within a region with DECIPHER missense constraint < 0.4 for the remaining genes [22].
    5. PM1_M for variants in a region with adequate, sufficient evidence from the literature supporting it as a hotspot or an important functional domain for the gene.

- **2F) PM4**
  - D: Protein length changes due to in-frame deletions/insertions in a nonrepeat region or stop-loss variants.
  - W: PM4_P; PM4_M
  - C: PM4, BP3 and PVS1 are mutually exclusive; variants should meet PM2_P or PM2_M for PM4 to be applied at any level
  - MA: The PM4 rule is not applicable to repetitive regions, defined as having more than 3 identical sequences (bases or sets of bases). Based on the principle that larger deletions/insertions in nonrepeating regions offer stronger evidence for pathogenicity, we have made the following modifications:
    1. PM4_P is employed for cases involving the insertion or deletion of 1 or 2 amino acids.
    2. PM4_M is employed for insertions or deletions of 3 or more amino acids.

- **2G) PM5**
  - D: Novel missense change at an amino acid residue where a different missense change determined to be pathogenic has been observed before.
  - W: PM5_P; PM5_M
  - C: PM1 and PM5 may overlap, and their simultaneous use requires caution. PM5 and PS1 are mutually exclusive
  - MA:﻿ PM5 is only used if there is sufficient supporting evidence that the molecular mechanism of pathogenicity is solely due to the missense effect; therefore, caution is recommended for variants with functional studies indicating aberrant splicing or a deleterious splicing prediction (SpliceAI delta score ≥ 0.8). The PM5 criterion is applied under the following conditions:
    1. PM5_P is applied for a variant that occurs in the same codon with a different missense variant that has been independently classified as likely pathogenic in only one previous report.
    2. PM5_M is applied if the different missense variants in the same codon have been independently classified as pathogenic in at least one previous report or likely pathogenic in two or more independent reports.

- **2H) PS1**
  - D: Same amino acid change (referred to as equivalent missense) as a previously established pathogenic variant regardless of nucleotide change
  - W: PS1_M; PS1_S
  - C: PS1 and PM5 are mutually exclusive
  - MA:﻿ PS1 is used only if there is sufficient supporting evidence that the molecular mechanism of pathogenicity is solely due to the missense effect; therefore, caution is recommended for variants with functional studies indicating aberrant splicing or a deleterious splicing prediction (SpliceAI delta score ≥ 0.8). The PS1 criterion is used according to the following conditions:
    1. PS1_M is applied if the equivalent missense variant has been independently classified as likely pathogenic in only one previous case.
    2. PS1_S is applied if the equivalent missense variant has been independently classified as pathogenic in at least one previous case or likely pathogenic in two or more independent cases.

- **2I) PVS1**
  - D: Null variant (nonsense, frameshift, canonical splice sites, initiation codon, single or multiexon deletion) in a gene where loss of function is a known mechanism of disease
  - W: PVS1_P; PVS1_M; PVS1_S; PVS1_VS
  - MA:﻿ The ClinGen haploinsufficiency score has been adopted as the primary tool for determining whether haploinsufficiency is the underlying disease mechanism for each gene [26]. In cases where ClinGen curation is unavailable, alternative sources such as the ExAC/gnomAD probability of LoF intolerance score (pLI > 0.9), observed/expected score (o/e < 0.35), OMIM, and available literature are utilized to assess the disease mechanism [25]. To guide the application of the PVS1 criterion, we adopted the decision tree guidelines proposed by Tayoun et al. [8] for appropriate modulation.

#### Case-level data

- **3A) BS2**
  - D: Observed in a healthy adult individual for a recessive (homozygous), dominant (heterozygous), or X-linked (hemizygous) disorder, with full penetrance expected at an early age.
  - W: BS2_S
  - MA: The BS2 criterion is employed according to the following specifications:
    1. BS2_S is used for in-house situations when individuals have a positive genotype but no corresponding phenotype for high-penetrance, early-onset conditions (such as cases with variants believed to be *de novo* in the proband but confirmed in healthy parents)[27].
    2. For cancer genes (Additional file 1: Table S1), BS2_S is used when the variant is found in homozygosity in at least one individual from control databases.
    3. For genes associated with rare AR or AD/AR diseases with complete penetrance, BS2_S is used when the variant is found in homozygosity in at least one individual from control databases.
    4. For genes associated exclusively with high-penetrance, pediatric-onset AD conditions (only genes included in the “green list” of the Severe Pediatric Disorders Database, available at panelapp.genomicsengland.co.uk/panels/921/), BS2_S is employed if the variant is present in at least five alleles.
    5. For genes associated with XLR or XL diseases, BS2_S is used when the variant is present in hemizygosity or homozygosity in at least one control subject.
    6. For genes associated exclusively with XLD conditions, BS2_S is used when the variant is found in heterozygosity in at least 5 control subjects.

- **3B) BS4**
  - D: Lack of segregation in affected members of a family.
  - W: BS4_S
  - C: BS4 and PP1 are mutually exclusive.
  - MA:﻿ The BS4_S criterion is utilized specifically for genes linked to high-penetrance, early-onset conditions where individuals possess a positive phenotype but lack a corresponding genotype.

- **3C) BP2**
  - D: Observed in *trans* with a pathogenic variant for a fully penetrant dominant gene/disorder or observed in *cis* with a pathogenic variant in any inheritance pattern
  - W: Not applied, except for curated variants from a recognized VCEP
  - MA: Due to the findings of recent molecular studies, which consistently indicate that phenotypes associated with recurrent variants can have broader manifestations or atypical findings, our group has made the decision not to utilize the BP2 criterion at present. Caution is advised when considering the use of this criterion that relies solely on the presence of a rare variant in *cis* or *trans* with a pathogenic variant, as we believe that it should not be considered as evidence for benignity.

- **3D) BP5**
  - D: Variant found in a patient with an alternate molecular cause for the disease.
  - W: BP5_P
  - MA:﻿ The BP5_P criterion may be employed exclusively when analyzing variants linked to AD highly penetrant, childhood-onset diseases. Specifically, it is only applied in cases where there is a clear alternate genetic cause (case with an alternate primary finding) for the observed phenotype and the variant is deemed unlikely to contribute to or modify the expressivity of the primary finding.

- **3E) PP1**
  - D: Cosegregation with disease in multiple affected family members in a gene definitively known to cause the disease
  - W: PP1_P; PP1_M; PP1_S
  - C: PP1 and BS4 are mutually exclusive. PP1, PS4 and PM3 may overlap, and their simultaneous use requires caution
  - MA: This criterion is used exclusively to count meioses of multiple affected family members (it may include more than one family if more than one affected individual is reported for every family). The number of meioses is used to modulate the weight, as follows:
    - 1 For AD conditions:
      - 1a) PP1_P: variant segregates with 3-4 meioses.
      - 1b) PP1_M: 5-6 meioses.
      - 1c) PP1_S: ≥ 7 meioses.
      2 For AR or AD/AR conditions:
      - 2a) PP1_P: variant segregates with 1 meiosis.
      - 2b) PP1_M: 2 meioses.
      - 2c) PP1_S: ≥ 3 meioses﻿.

- **3F) PP4**
- D: Patient’s phenotype or family history is highly specific for a disease with a single genetic etiology.
- W: PP4_P; PP4_S
- MA:﻿ To apply the PP4 criterion, the following conditions must be met: a) the test performed must be comprehensive, encompassing all relevant genes and molecular mechanisms, including copy number analysis, that could potentially contribute to the observed phenotype; b) the variant should be rare in the absence of other candidate disease-causing variants; and c) the family history should align with the expected pattern of inheritance. The PP4 criterion is then utilized in the following manner:
  1. PP4_P is applied when all these circumstances are satisfied.
  2. If there is additional clinical evidence, such as pathognomonic muscle biopsy, biochemistry, or an 'exclusive' clinical diagnosis, the criterion can be upgraded to PP4_S.

- **3G) PM3**
  - D: For recessive disorders, detected in *trans* with a pathogenic variant.
  - W: PM3_P; PM3_M; PM3_S; PM3_VS
  - C: PM3, PS4 and PP1 may overlap, and their simultaneous use requires caution.
  - MA: ﻿PM3 is the primary criterion used to count probands for AR conditions and has been modified to a scoring system (SS) that incorporates clinical reports from the literature to modulate the PM3 weight. For every unrelated affected individual (eventually including the assessed proband), a score of 1.0 is applied when the variant is observed in *trans* with a known pathogenic or likely pathogenic variant, a score of 0.5 if the phase is unknown, and a score of 0.5 if the variant is found in homozygosity (downgraded to 0.25 if the parents are consanguineous). PM3 may be used in the following situations (Additional file 1: Table S3):
    1. PM3_P for SS ≥ 0.5.
    2. PM3_M for SS ≥ 1.0.
    3. PM3_S for SS ≥ 2.0.
    4. PM3_VS for SS ≥ 4.0.

- **3H) PM6**
  - D: Assumed to be de novo, but without confirmation of paternity and maternity.
  - W: PM6_P; PM6_M; PM6_S; PM6_VS
  - C:﻿ PM6 and PS2 may overlap, and their simultaneous use requires caution.
  - MA: PM6 is the criterion used to count assumed de novo events and has been modified to an SS that modulates its weight, according to the quantitative approach proposed by ClinGen. For every unrelated affected individual (eventually including the assessed proband), a score of 1.0 is applied when the variant is assumed to be de novo in a phenotype highly specific for the gene, a score of 0.5 if the phenotype is consistent with the gene but is not highly specific, a score of 0.25 if the phenotype is consistent with the gene but is not highly specific and has high genetic heterogeneity, and a score of zero if the phenotype is not consistent with the gene. PM6 may be used in the following situations (Additional file 1: Table S4):
    1. PM6_P for SS ≥ 0.5.
    2. PM6_M for SS ≥ 1.
    3. PM6_S for SS ≥ 2.
    4. PM6_VS for SS ≥ 4.

- **3I) PS2**
  - D: De novo (both maternity and paternity confirmed) in a patient with the disease and no family history.
  - W: PS2_P; PS2_M; PS2_S; PS2_VS
  - C: PS2 and PM6 may overlap, and their simultaneous use requires caution.
  - MA:﻿ PS2 is the criterion used to count confirmed de novo events and has been modified to an SS that modulates its weight, according to the quantitative approach proposed by ClinGen. For every unrelated affected individual (eventually including the assessed proband), a score of 2.0 is applied when the variant is assumed to be de novo in a phenotype highly specific for the gene, a score of 1.0 if the phenotype is consistent with the gene but is not highly specific, a score of 0.50 if the phenotype is consistent with the gene but is not highly specific and has high genetic heterogeneity, and a score of zero if the phenotype is not consistent with the gene. PM6 may be used in the following situations (Additional file 1: Table S5):
    1. PS2_P for SS ≥ 0.5.
    2. PS2_M for SS ≥ 1.
    3. PS2_S for SS ≥ 2.
    4. PS2_VS for SS ≥ 4.

- **3 J) PS4**
  - D: The prevalence of the variant in affected individuals is significantly increased compared to the prevalence in controls.
  - W: PS4_P; PS4_M; PS4_S
  - C: PS4, PM3 and PP1 may overlap, and their simultaneous use requires caution.
  - MA:﻿ This criterion has been modified to incorporate clinical reports from the literature. It may be used in the following situations (Additional file 1: Table S6):
    1. PS4_S is applied when case‒control studies demonstrate a statistically significant increased frequency of a variant in affected individuals compared to controls (odds ratio or relative risk > 5 and confidence interval not including 1).
    2. PS4_S is applied for known founder variants (pathogenic variant observed at high frequency in a specific population).
    3. PS4_S is applied for variants curated as pathogenic by recognized ClinGen expert panels.
    4. PS4 is the criterion for counting probands for AD conditions and may be applied for very rare variants that fulfill the PM2_M or PM2_P criteria; are associated with dominant conditions; and are observed in previously described, unrelated patients with a confirmed phenotype with the following weights:
    - 4a) For cancer genes (Additional file 1: Table S1):
    - 4a1) PS4_P for 2-5 probands.
    - 4a2) PS4_M for 6-9 probands.
    - 4a3) PS4_S for ≥ 10 probands.
    - 4b) For the remaining genes:
    - 4b1) PS4_P for 1-2 probands.
    - 4b2) PS4_M for 3-4 probands.
    - 4b3) PS4_S for ≥ 5 probands.

#### Functional and computational data

- **4A) BS3**
  - D: Well-established in vitro or in vivo functional studies show no damaging effect on protein function or splicing.
  - W: BS3_P; BS3_M; BS3_S
  - C: BS3 and PS3 are mutually exclusive.
  - MA:﻿ For BS3, we adhered to the recommendations and structured approach proposed by Brnich et al. [9] for the assessment of functional assays in variant interpretation and the use of different levels of strength according to assay validation.

- **4B) PS3**
  - D: Well-established in vitro or in vivo functional studies supportive of a damaging effect on the gene or gene product.
  - W: PS3_P; PS3_M; PS3_S; PS3_VS
  - C: PS3 and BS3 are mutually exclusive.
  - MA:﻿ For PS3, we adhered to the recommendations and structured approach proposed by Brnich et al. [9]. The proband´s personal functional assays must not be considered for PS3 use (for patient-derived evidence, consider the use of PP4).

- **4C) BP4**
  - D: Multiple lines of computational evidence suggest no impact on genes or gene products.
  - W: BP4_P
  - C: BP4, PP3 and BP7 are mutually exclusive.
  - MA:﻿ The BP4_P criterion is applied according to the following specifications:
    1. For a subset of 78 genes with specific recommendations from ClinGen, the REVEL thresholds for missense variants and SpliceAI scores for noncoding variants are outlined in Additional file 1: Table S7.
    2. For missense variants found in genes without specific recommendations, a REVEL score < 0.4 is utilized [28].
    3. For noncoding variants in genes without recommendations, a SpliceAI delta score ≤ 0.2 is employed.

- **4D) PP3**
  - D: Multiple lines of computational evidence support a deleterious effect on the gene or gene product.
  - W: PP3_P
  - C: PP3, BP4 and BP7 are mutually exclusive.
  - MA:﻿ The PP3_P criterion is applied according to the following specifications:
    1. For a subset of 78 genes with specific recommendations from ClinGen, the REVEL thresholds for missense variants and SpliceAI scores for noncoding variants are outlined in Additional file 1: Table S7.
    2. For missense variants found in genes without specific recommendations, a REVEL score > 0.7 is utilized [28].
    3. For noncoding variants in genes without recommendations, a SpliceAI delta score ≥ 0.8 is employed﻿.

#### Reputable source

- **5A) PP5**
  - D: A reputable source recently reports the variant as pathogenic, but the evidence is not available to the laboratory to perform an independent evaluation.
  - W: Not applied.
  - MA: This criterion has been discontinued [7]﻿.
- **5B) BP6**
  - D: A reputable source recently reports the variant as benign, but the evidence is not available to the laboratory to perform an independent evaluation.
  - W: Not applied.
  - MA:﻿ This criterion has been discontinued [7].

### Combining criteria for variant classification

We adopted the same classifications for all variants curated, assessed and classified through a ClinGen-approved Variant Curation Expert Panel. All other variants go through the systematic process described above of collecting evidence from multiple sources, translating these different pieces of evidence into ACMG/AMP criteria, refining their strength levels when appropriate and then combining them all for the final classification of each variant. Following the ACMG/AMP guidelines, we have established the following:

- 1 Benign variants require one of the following:
  - 1A) Stand-alone BA1.
  - 1B) ≥ 2 Strong.
- 2 Likely benign variants must satisfy one of the following:
  - 2A) 1 Strong and 1 Supporting.
  - 2B) ≥2 Supporting.
- 3 Likely pathogenic variants require one of the following:
  - 3A) 1 Very Strong AND 1 Moderate.
  - 3B) PVS1_VS AND PM2_P.
  - 3C) 1 Strong AND 1–2 Moderate.
  - 3D) 1 Strong AND ≥2 Supporting.
  - 3E) ≥3 Moderate.
  - 3F) 2 Moderate AND ≥2 Supporting.
  - 3G) 1 Moderate AND ≥4 Supporting.
- 4 Pathogenic variants require one of the following:
  - 4A) 1 Very Strong AND ≥1 Strong.
  - 4B) 1 Very Strong AND ≥2 Moderate.
  - 4C) 1 Very Strong AND 1 Moderate AND 1 Supporting.
  - 4D) 1 Very Strong AND ≥2 Supporting.
  - 4E) ≥2 Strong.
  - 4F) 1 Strong AND ≥3 Moderate.
  - 4G) 1 Strong AND 2 Moderate AND ≥2 Supporting.
  - 4H) 1 Strong AND 1 Moderate AND ≥4 Supporting.

#### Bioinformatics and automated classification

Our service uses the Varstation ® Platform (version 2.0, São Paulo, Brazil, https://varsomics.com/varstation/) for variant analysis, interpretation and classification. Varstation is a user-friendly, cloud-based software tool for analyzing human genetic variation from NGS data [29]. Genomic workflows are created, customized and automated in the cloud environment using Cromwell as a workflow management system at the AWS cloud infrastructure.

Among several functionalities, Varstation provides a semiautomated variant classification workflow according to ACMG/AMP and ClinGen guidelines and our own recommendations (described in this article). It collects information from more than 40 public databases (e.g., gnomAD, ClinVar, dbSNP, OMIM, in silico predictors) and automatically interprets 17 criteria based on a decision tree framework: PVS1, PS1, PM1, PM2, PM4, PM5, PP2, PP3, PP5, BA1, BS1, BS2, BP1, BP3, BP4, BP6, and BP7. Although PP5 and BP6 use is discouraged by ClinGen [7], these rules are still evaluated by Varstation. The remaining criteria (PS2, PS3, PS4, PM3, PM6, PP1, PP4, BS3, BS4, BP2, BP5) require manual curation by the user due to lack of data (e.g., clinical history, family segregation, functional studies). For mutually exclusive rules (e.g., BA1, BS1 and PM2), the strongest rule is chosen.

For each evaluated criterion, all supporting evidence is fully described, allowing the reduction of sequence variant interpretation time and helping health care professionals understand and prioritize potential disease-causing variants. Users can also reinterpret all 28 criteria and change variant clinical significance depending on their own evidence and expertise.

Varstation is constantly updated as new publications and data sources are published. Gene-specific recommendations (ClinGen VCEPs) and the Bayesian model for variant classification are implemented in Varstation version 3.0. Varstation is freely available for academic and nonprofit users at https://varsomics.com/varstation/.

## Discussion

The field of variant interpretation has undergone significant advancements and refinements in the last decade. The adoption of standardized guidelines, such as the ACMG/AMP, has played a crucial role in achieving consistency and uniformity in variant classification among scientists from laboratories all over the world. These guidelines provide a systematic approach by considering specific criteria and evidence types. However, the continuous evolution of genetics research and the increasing complexity of genomic data interpretation have required updates to these guidelines. The development of our internal consensus and standards for constitutional sequence variant classification adapted to some specific characteristics of the Brazilian population further contributes to the continuous refinement of variant classification practices.

One of the main weaknesses of the original ACMG/AMP guidelines is that they relied mainly on expert opinion and empirical data [2], which are generally considered a low level of scientific evidence. To address this limitation, various modifications to the original guidelines have been proposed over the years. These changes have resulted, on the one hand, in a more evidence-based approach and reduced reliance on expert opinion-based criteria. On the other hand, we observed a less unified process for incorporating these modifications. Several reasons may explain the latter. First, there is a lack of a single comprehensive document that incorporates all proposed modifications. Second, the increasing complexity of gene-based rules and their exceptions poses a significant barrier to timely adoption by all laboratories. Finally, disagreements among different services regarding the use of various criteria have led to further fragmentation of the classification process.

Our team believes that a reunification of the variant classification process would benefit from a shift toward more quantitative criteria and the adoption of Bayesian statistical reasoning. Tavtigian et al. [5] incorporated the ACMG/AMP guidelines into a Bayesian framework, evaluated each of the 18 combining rules outlined in the guidelines for consistency and determined whether the posterior probability (Post_P) for each scenario fell within the expected range for its classification. Overall, the evaluation of the combining rules within a Bayesian framework demonstrated high consistency with the ACMG/AMP guidelines.

Only two of the 18 combining rules were mathematically inconsistent with the overall framework. The first inconsistent rule, “ ≥ 2 Strong = Pathogenic”, resulted in a Post_P of 0.975, which falls within the likely pathogenic range (0.90–0.99) rather than the pathogenic range (> 0.99). The second inconsistent rule, “1 Very Strong AND 1 Moderate = Likely pathogenic”, resulted in a Post_P of 0.994, which falls within the pathogenic range (> 0.99). This latter combination is commonly used for novel loss-of-function variants that have one very strong piece of evidence and one moderate piece of evidence. It is also important to note that the use of BA1 as absolute evidence that a variant is benign is contrary to Bayesian reasoning [5].

While we wait for a broader adoption of this Bayesian approach, we have proactively incorporated these important concepts into our internal criteria and will soon start providing both qualitative variant classification (e.g., pathogenic, benign, VUS) and the Post_P score. We believe that this approach represents an important step forward to a more unified and standardized variant classification process, which will ultimately benefit laboratories, clinicians, and patients.

These internal standards, along with the original ACMG/AMP guidelines, are exclusively focused on constitutional (frequently referred to as “germline”) sequence variants associated with monogenic disorders and do not intend to include structural, mitochondrial or somatic variants or oligogenic or complex inheritance. For those situations, specific guidelines have been proposed, such as a joint consensus for somatic variant curation [30] and for copy-number variants [31].

In the era of artificial intelligence, there has been a noticeable shift in the importance placed on functional assays that test variant impact [9]. These assays, which have traditionally been critical in assessing the functional consequences of variants, have taken a back seat with the emergence of meta-predictors. Meta-predictors, utilizing machine learning algorithms and integrating diverse genomic data, have gained prominence, and studies are suggesting higher weights (moderate to strong evidence levels) based on their predictive scores [13, 15]. However, it is important to note that this shift toward computational approaches is not unanimously accepted. Given the concerns raised within our workgroup, we have chosen to exercise caution and await further evidence and validation before fully embracing this approach.

## Conclusions

This manuscript discusses the most recent 2023 version of the Hospital Israelita Albert Einstein Standards for Constitutional Sequence Variants Classification. These updated standards incorporate modifications proposed by leading genetics societies, ClinGen and our established internal consensus. These efforts from our workgroup aim to enhance the reliability and uniformity of variant classification.

## Methods

### Albert Einstein hospital workgroup

In 2022 and 2023, a workgroup consisting of medical geneticists, molecular biologists, and bioinformaticians from HIAE was established to review the literature on best practices and standards for sequence variant classification and develop an internal consensus. The workgroup focused solely on sequence variants (single-nucleotide variants and small indels).

This study adheres to the principles of the Declaration of Helsinki and is in accordance with Brazilian and HIAE statutory requirements. Ethics committee approval was not necessary since the study did not involve live subjects or sensitive data.

### Supplementary Information

**Additional file 1. Supplementary Table S1-Cancer genes:** The process of criteria modulation and variant classification for cancer genes has particular specifications in our service that differ from our general rule. Below is a compilation of the cancer genes to which these specifications apply.

## Data Availability

All data are provided as “Additional file 1 ”.

## Abbreviations

A: Stand-alone
ABraOM: Online Archive of Brazilian Mutations
AD: Autosomal dominant
ACMG: American college of medical genetics and genomics
AFR: African/African American
AMP: Association for Molecular Pathology
AMR: American Admixed/Latino
AR: Autosomal recessive
AD/AR: Autosomal dominant and autosomal recessive
B: Benign
C: Conflicts with other criteria
ClinGen: Clinical genome resource
dbSNP: Single-nucleotide polymorphism database
DECIPHER: Database of chromosomal imbalance and phenotype in humans using ensembl resources
EAS: East Asian
gnomAD: Genome aggregation database
HIAE: Hospital Israelita Albert Einstein
M: Moderate
MA: Modifications and adaptations
NFE: Non-Finnish European
NGS: Next-generation sequencing
OMIM: Online Mendelian Inheritance in Man
P: Supporting
Post_P: Posterior probability
Pt: Pathogenic
S: Strong
SAS: South Asian
SS: Scoring system
VCEP: Variant curation expert panel
VS: Very strong
VUS: Variant of uncertain significance
W: Weight modulations
XL: X-linked
XL-D: X-linked dominant
XL-R: X-linked recessive
